# Extracranial meningiomas concurrently found in the lung and vertebral bone: a case report

**DOI:** 10.1186/s13256-018-1826-5

**Published:** 2018-09-28

**Authors:** Hiromi Tamura, Yasushi Otani, Takashi Iwazawa, Masafumi Kashii, Hiroka Ando, Reiko Doi, Shiro Adachi

**Affiliations:** 1 Department of Pathology, Toyonaka Municipal Hospital, Shibahara-cho 4-14-1, Toyonaka, Osaka 560-8565 Japan; 2 Department of Respiratory Medicine, Toyonaka Municipal Hospital, Shibahara-cho 4-14-1, Toyonaka, Osaka 560-8565 Japan; 3 Department of Surgery, Toyonaka Municipal Hospital, Shibahara-cho 4-14-1, Toyonaka, Osaka 560-8565 Japan; 4 Department of Orthopedics, Toyonaka Municipal Hospital, Shibahara-cho 4-14-1, Toyonaka, Osaka 560-8565 Japan

**Keywords:** Extracranial meningioma, Lung, Vertebral bone

## Abstract

**Background:**

Primary pulmonary meningiomas are very rare, and primary intraosseous meningiomas outside the head and neck region have not yet been reported. We report an extremely unusual case of concurrent meningiomas arising in the pulmonary parenchyma and vertebral bone.

**Case presentation:**

A 40-year-old Asian woman presented with a destructive lesion of the lumbar vertebral bone and a small nodule in the right lung. Five years later, both lesions slightly increased in size. To evaluate both the pulmonary and vertebral lesions, video-assisted thoracic surgery and curettage of the lytic lesion were performed. Both lesions showed similar histopathological findings corresponding to an intracranial meningioma of World Health Organization grade 1. The patient made good postoperative progress and remained free from disease at 41 months after the operation.

**Conclusions:**

Our patient presented with almost synchronous pulmonary and lumbar vertebral intraosseous meningiomas. Regarding the relationship between the two lesions, there are two possibilities: Independent tumors occurred coincidentally or the primary pulmonary meningioma metastasized to the vertebral bone despite its bland morphology. It is important to keep in mind the exceptionally rare condition of extracranial meningioma.

## Background

Meningiomas are relatively common primary central nervous system tumors, comprising approximately 25% of all intracranial neoplasms [1]. Primary extracranial and extraspinal meningiomas are rare neoplasms, with the vast majority located in the head and neck region [2]. Primary pulmonary meningiomas are even less common, and almost all of the reported cases have been benign [3–12]. There have been no reports of primary intraosseous meningiomas outside the head and neck region. We report an extremely unusual case of concurrent meningiomas arising in the pulmonary parenchyma and vertebral bone in a 40-year-old woman. The patient showed no evidence of a tumor on imaging examinations of the head and neck. The clinicopathological features of the patient and immunoprofile of the tumor are presented.

## Case presentation

**Fig. 1:**
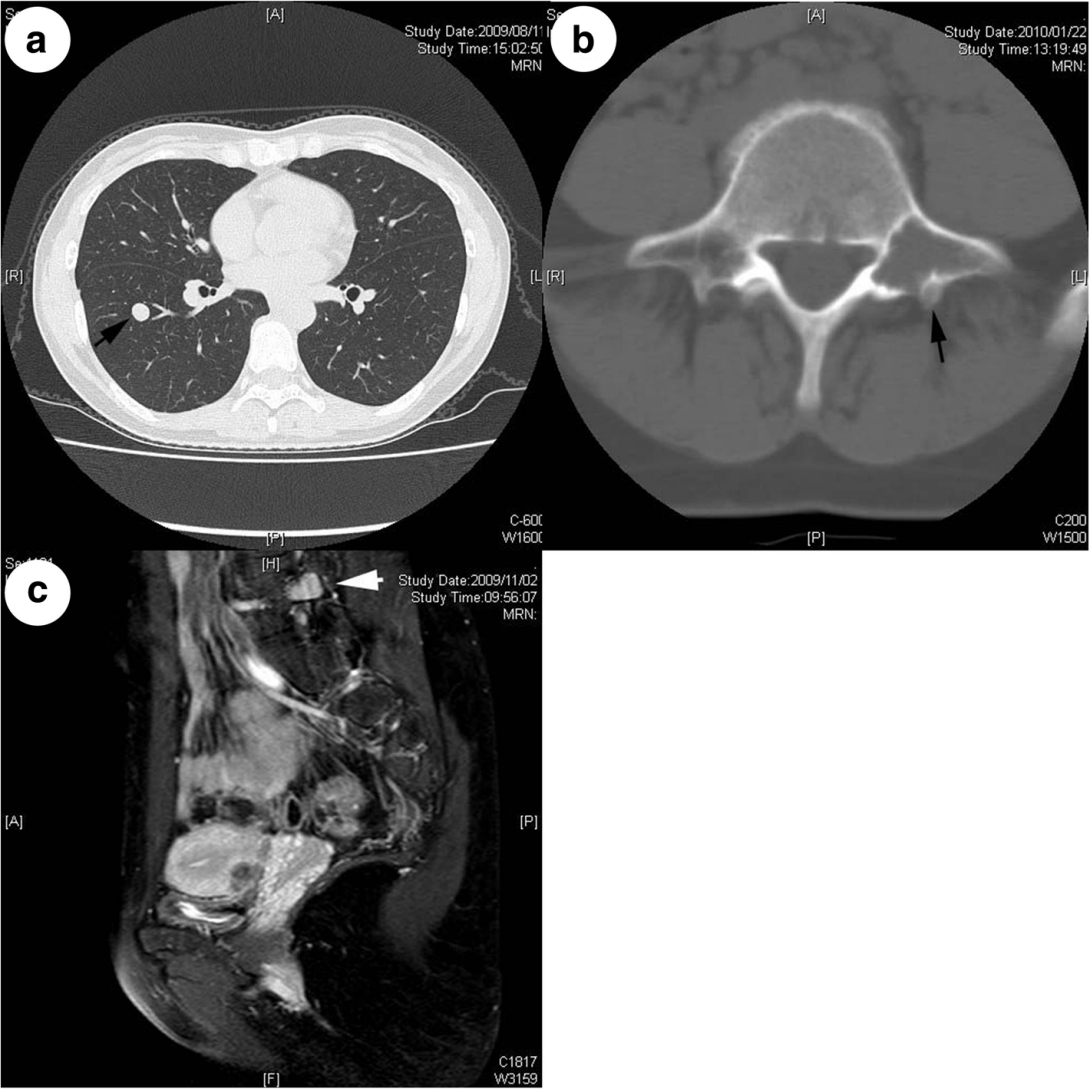
**a** Computed tomographic scan showing a well-circumscribed nodule in the right lung (arrow). **b** Magnetic resonance (MR) image showing a lytic lesion in the left transverse process in the fifth lumbar vertebra (arrow). The lesion appeared to erupt to the posterior surface of the transverse process. No mass lesion was found in the spinal canal. **c** Sagittal MR image of the lumbar region. A mass lesion was situated posterior to the lumbar vertebral column (arrow)

A 40-year**-**old Asian woman who had been in her usual state of good health was incidentally found to have a small nodule in the lower lobe of her right lung during a regular medical checkup. The patient was seen in the department of medicine of our hospital for further evaluation. The patient’s family history was noncontributory, and a physical examination revealed no abnormalities. The results of laboratory studies were either within normal limits or negative. A computed tomographic (CT) scan revealed a lytic lesion of the fifth lumbar vertebral bone, as well as a small nodule (1.2 cm in diameter) in the lower lobe of the right lung (Fig. 1a). The vertebral lesion was situated on the left transverse process and was separated from the spinal canal (Fig. 1b, c). Biopsy specimens of both lesions were histologically similar and showed spindle cell neoplasms with a bland appearance; however, we could not make a definitive diagnosis, owing to the small size of the samples. The patient was placed under close observation with suspicion of multiple metastases from an unknown primary tumor.

Five years later, a CT scan indicated that the pulmonary nodule had slightly increased in size. Magnetic resonance (MR) imaging also revealed an increase in the size of the lytic lesion in the left transverse process of the fifth lumbar vertebra. To evaluate both the pulmonary and vertebral lesions, video-assisted thoracic surgery for the pulmonary lesion and curettage of the lytic bone lesion were performed. The orthopedic surgeons found that the tumor of the fifth left transverse process was exposed on the posterior surface but not on the anterior surface. They also found that the vertebral lesion was completely confined within the vertebral bone and was not connected to the spinal canal. During the clinical course (105 months), no significant findings other than the pulmonary and vertebral lesions were found, even with imaging examinations, including CT and MR imaging. The patient made good postoperative progress and remained free of disease at 41 months after the operation.

**Fig. 2:**
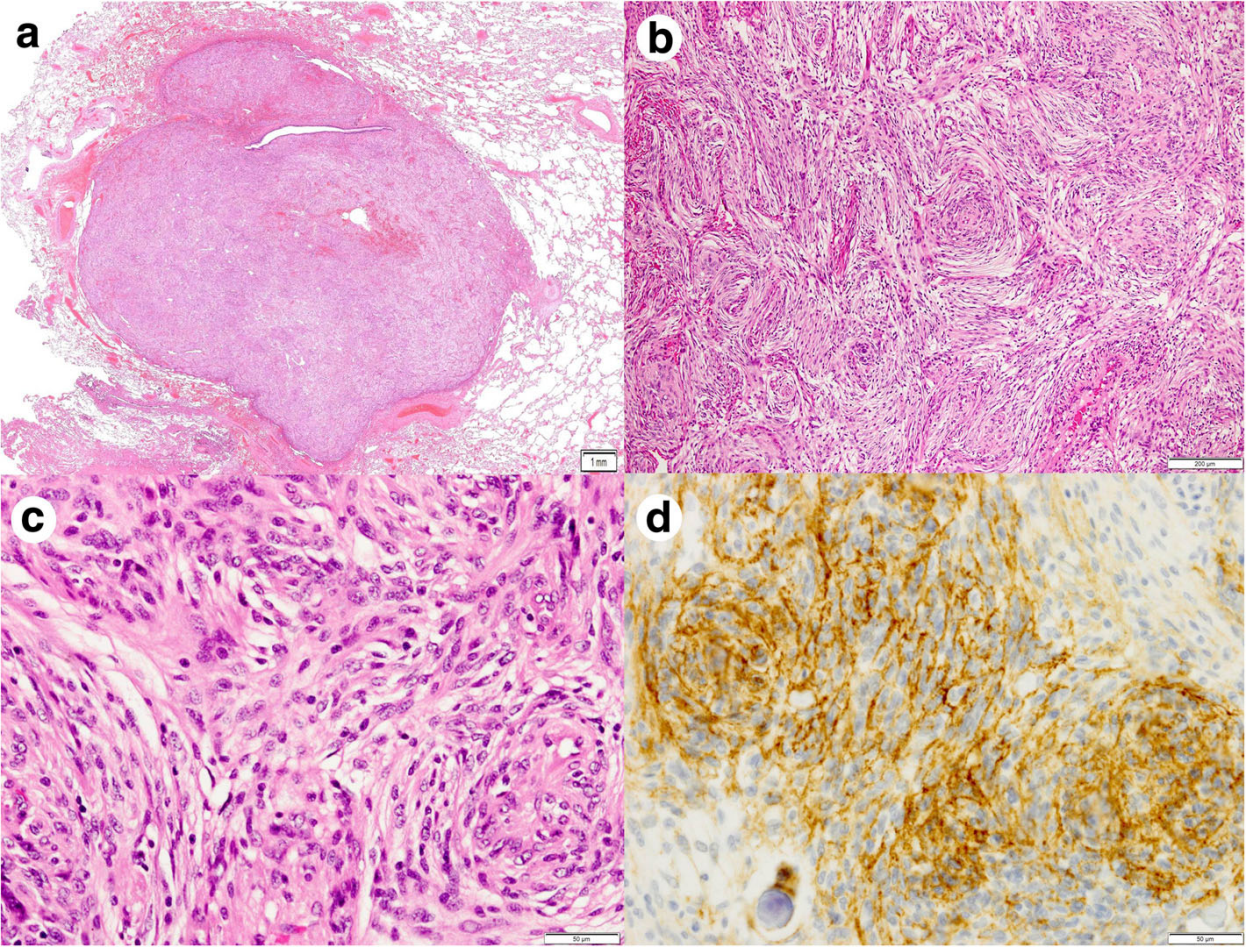
**a** Panoramic view of the pulmonary nodule. A bronchiole and terminal air spaces were scattered in the tumor. **b** No necrotic foci were observed. The tumor showed relatively monotonous proliferation of eosinophilic spindle cells. The tumor cells were arranged in a fascicular or slightly whorled pattern. **c** The cells were uniform, the nuclei exhibited minimal pleomorphism without nucleoli, and intranuclear cytoplasmic inclusions were easily identified. Mitotic figures were not observed. **d** The cells were immunopositive for epithelial membrane antigen

**Fig. 3:**
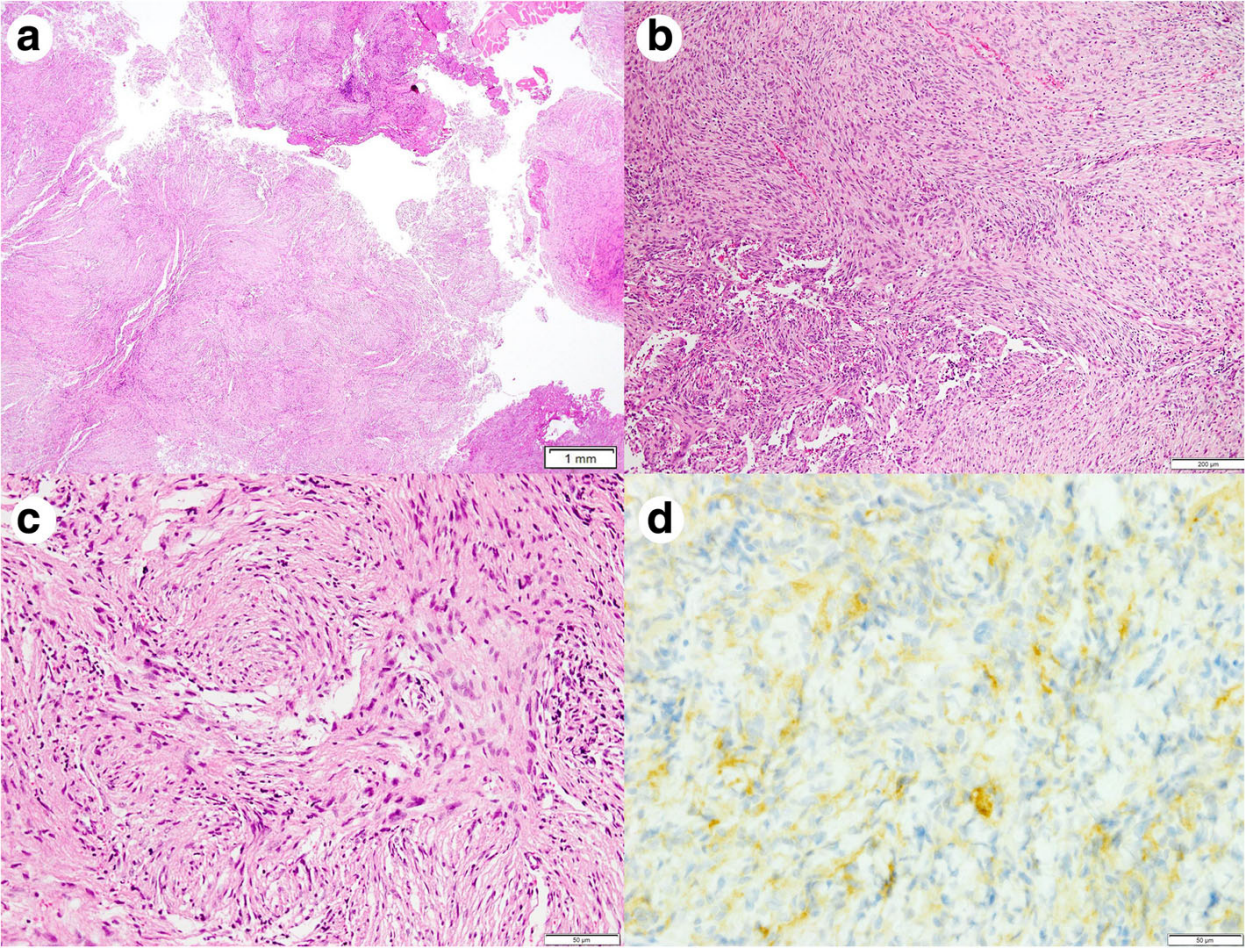
**a** Histological appearance of the tumor from the transverse process. **b** and **c** Closer observation of the tumor. The morphological findings were identical to those of the pulmonary lesion (Fig. 2). **d** The cells were also reactive for epithelial membrane antigen

The resected specimen of the lung contained a well-circumscribed solid lesion 13 mm in diameter. It was composed of fascicular architecture of bland spindle or polygonal cells (Fig. 2a–c). Neither necrotic foci nor mitotic figures were observed. The tumor was immunopositive for epithelial membrane antigen (Fig. 2d), D2-40, progesterone receptor, vimentin, and S100 but negative for alpha-smooth muscle actin. Although curettage specimens from the vertebral bone were fragmented, the morphological and immunohistochemical findings were identical to those of the pulmonary lesion (Fig. 3a–d). The tumor was shown to be a transitional meningioma on histological examination, including immunohistochemical analyses (Table 1).

**Table 1:** Immunohistochemical profiles of both pulmonary and vertebral lesions

Antibody	Pulmonary lesion	Vertebral lesion
Anti-epithelial membrane antigen	Focal/moderate	Focal/moderate
D2-40	Diffuse/strong	Diffuse/strong
Anti-progesterone receptor	Diffuse/strong	Diffuse/strong
Anti-vimentin	Diffuse/strong	Diffuse/strong
Anti-S100 antigen	Focal/moderate	Focal/moderate
Anti-alpha-smooth muscle actin	Negative	Negative
Ki-67 index	1.52%	1.84%

The Ki-67 labeling index was measured using the Ventana iScan HT scanner (Roche Diagnostics, Indianapolis, IN, USA)

## Discussion

The occurrence of ectopic meningiomas is well known [2]. Although pulmonary meningiomas have been documented, they are rare [3–12]. Our patient presented with synchronous pulmonary and lumbar vertebral intraosseous meningiomas.

We postulate two possibilities concerning the relationship between these lesions. First, it is possible that the lesions happened to coincide. Although intraosseous meningioma can occur as a primary tumor exclusively in the head and neck region [13], there have been no reports convincingly describing primary meningioma in the bone outside these areas. Meningioma originating from the spinal meninges can invade the vertebral bone as if it had arisen in the vertebral bone. However, in our patient, both imaging examinations and intraoperative observations by orthopedists clearly excluded the presence of a spinal meningeal tumor. If each tumor had occurred independently, it would follow that intraosseous meningiomas could arise outside the head and neck region. Second, the pulmonary meningioma may have metastasized to the vertebral bone, or the reverse scenario may have been present. Because no other lesions were found despite thorough examinations during the follow-up period of 41 months, it is unlikely that the undetectable primary tumor metastasized to the lung and vertebral bone. The histopathological and immunohistochemical features of the vertebral bone lesion were identical to those of the pulmonary lesion. Although almost all meningiomas are benign, there have been reported cases of extracranial malignant meningiomas. Both meningiomas in our patient corresponded to a World Health Organization grade 1 intracranial meningioma. These histopathological features generally suggest benignancy; however, metastasis is a well-documented phenomenon, even in intracranial meningiomas with grade 1 histology [14]. Thus, it is not surprising that the meningioma with grade 1 histology in our patient metastasized to a distant organ. Although it is not always easy to determine the origin of the primary lesion, it appeared that the lung rather than the vertebral bone was the primary organ, because no cases of primary meningioma in the bone outside the head and neck region have been recorded. Among the 40 cases of primary pulmonary meningioma reported to date, only 2 have been of indisputable primary malignant meningioma [9, 11]. In both cases, the tumors had anaplastic morphology. If the pulmonary meningioma had metastasized to the vertebral bone, it would have been surprising that primary pulmonary meningioma without significant atypia had metastatic potential.

The differential diagnosis of meningioma includes a number of lesions composed of bland-looking eosinophilic spindle cells. When examining the entire lesion, the diagnosis may be straightforward if the pathologists are aware of the presence of the extracranial meningioma. However, when biopsy specimens are inadequate, diagnosis can be challenging. Primary leiomyoma, which arises in the bronchial wall, is also composed of bland and eosinophilic spindle cells [15]. The location of the lesion will help in discrimination. Metastasizing leiomyoma, the histological features of which are identical to those of primary leiomyoma, can be found in premenopausal women, as in our patient [16]. A history of hysterectomy for uterine leiomyoma is essential for the diagnosis. Intrapulmonary thymoma may also enter into the differential diagnosis [17]. The intralesional fibrous septa and/or intralesional lymphocytic infiltrate, which typify thymoma, will facilitate correct diagnosis. Solitary fibrous tumors appear not only as pleural lesions but also as intrapulmonary (inverted) lesions [18]. Branched intralesional blood vessels resembling moose antlers and/or a so-called patternless pattern of the tumor cells are absent in meningiomas. Immunohistochemical examinations will clearly discriminate the aforementioned lesions when pathologists consider the possibility of meningioma (Table 2).

**Table 2:** Differential diagnosis of meningioma: immunohistochemical findings [16, 19, 20]

Antibody	Meningioma	Leiomyoma	Thymoma (spindle cell type)	Solitary fibrous tumor
Anti-epithelial membrane antigen	+	–	–	–
Anti-vimentin	+	+	+	+
Anti-S100 antigen	Occasionally +	–	–	–
Anti-alpha smooth muscle actin	–	+	Rarely +	–
CD34	–	–	–	+
Anti-pan-cytokeratin (AE1/AE3)	–	–	+	–

## Conclusions

In summary, we describe an extremely unusual case of a patient with concurrent meningiomas arising in the pulmonary parenchyma and lumbar vertebral bone. Although the history of these tumors cannot be clearly determined, it is important to recognize that such an unusual situation can occur.

## Abbreviations

CT: Computed tomographic
MR: Magnetic resonance
